# Tissue-specific effects of temperature on proteasome function

**DOI:** 10.1007/s12192-020-01107-y

**Published:** 2020-04-18

**Authors:** Johanna Pispa, Olli Matilainen, Carina I. Holmberg

**Affiliations:** 1 Medicum, Department of Biochemistry and Developmental Biology, Faculty of Medicine, University of Helsinki, Helsinki, Finland; 2 Institute of Biotechnology, University of Helsinki, Helsinki, Finland

**Keywords:** Ubiquitin-proteasome system (UPS), Ambient temperature, *Caenorhabditis elegans*, Tissue specificity, Stress

## Abstract

Variation in ambient growth temperature can cause changes in normal animal physiology and cellular functions such as control of protein homeostasis. A key mechanism for maintaining proteostasis is the selective degradation of polyubiquitinated proteins, mediated by the ubiquitin-proteasome system (UPS). It is still largely unsolved how temperature changes affect the UPS at the organismal level. *Caenorhabditis elegans* nematodes are normally bred at 20 °C, but for some experimental conditions, 25 °C is often used. We studied the effect of 25 °C on *C. elegans* UPS by measuring proteasome activity and polyubiquitinated proteins both in vitro in whole animal lysates and in vivo in tissue-specific transgenic reporter strains. Our results show that an ambient temperature shift from 20 to 25 °C increases the UPS activity in the intestine, but not in the body wall muscle tissue, where a concomitant accumulation of polyubiquitinated proteins occurs. These changes in the UPS activity and levels of polyubiquitinated proteins were not detectable in whole animal lysates. The exposure of transgenic animals to 25 °C also induced ER stress reporter fluorescence, but not the fluorescence of a heat shock responsive reporter, albeit detection of a mild induction in *hsp-16.2* mRNA levels. In conclusion, *C. elegans* exhibits tissue-specific responses of the UPS as an organismal strategy to cope with a rise in ambient temperature.

**Electronic supplementary material:**

The online version of this article (10.1007/s12192-020-01107-y) contains supplementary material, which is available to authorized users.

## Introduction

Temperature is a key environmental factor that affects various aspects of animal physiology. The impact of temperature has mainly been studied in the context of thermal stress of either extreme heat or cold, e.g. in model organisms such as *Drosophila melanogaster*, mice, and fish (Christians and Benjamin 2005; Haslbeck et al. 2012; Soyano and Mushirobira 2018; Yu et al. 2015). Studies on the effects caused by smaller changes in ambient temperature are less common. The ectotherm nematode *Caenorhabditis elegans* is normally grown in the laboratory at 20 °C, but experimental conditions may require growth within a temperature range from 15 to 25 °C. For example*,* life span analyses are often performed at 25 °C for practical reasons. Despite the common experimental use of 25 °C, the consequences of the increased temperature on *C. elegans* physiology are not completely understood, in particular its effects on protein degradation.

Although most *C. elegans* strains can be bred at 25 °C, it is obvious that this temperature is not optimal for growth and can be stressful for the organism. Both development and ageing progress faster, and the progeny number is reduced at 25 °C (Klass 1977). Furthermore, the metabolic rate increases as measured by carbon dioxide production (Van Voorhies and Ward 1999). Some recent studies have compared the effects of 20 °C and 25 °C on expression by using either global mRNA-sequencing (Gómez-Orte et al. 2018; Schott et al. 2014) or phosphopeptide identification (Huang et al. 2018), and, e.g., upregulation of the innate immune response occurs at 25 °C (Gómez-Orte et al. 2018). How such small, yet meaningful, changes in ambient temperature are reflected in proteostasis in vivo is still a largely unanswered question. The cell utilises two main pathways for protein degradation: the ubiquitin-proteasome system (UPS) and autophagy-lysosome system. In the UPS, the key component is the proteasome, a large protein complex containing a catalytic core particle, CP or 20S, which consists of two heptameric rings of alpha and two of beta subunits. The core can be flanked by either one or two regulatory particles, RPs or 19S, which are responsible for the recognition and unfolding of polyubiquitinated proteins (Finley et al. 2016). In the killifish species *Nothobranchius rachovii*, a linear inverse correlation is seen in muscle tissue between proteasome activity and ambient temperature, without a detectable change in proteasome amount (Lu and Hsu 2015). Also, raising the room temperature of mice housed at 22 to 30 °C decreases in vitro measured proteasome activity from brown adipose tissue (Bartelt et al. 2018). At a gene expression level, results are more variable. *Drosophila melanogaster* exposed to a high ambient temperature shows upregulation of some of the proteasome CP and RP subunits (Kristensen et al. 2016). Similarly, in corals, upregulation of proteasome-related genes is one of the first transcriptional responses to rising temperatures (Maor-Landaw et al. 2014; Traylor-Knowles et al. 2017). In contrast, several proteasome subunit mRNAs are downregulated in *C. elegans* grown for several generations at 25 °C whereas no effect is observed on ubiquitin transcript levels (Gómez-Orte et al. 2018). Interestingly, overexpression of a proteasome RP subunit, *rpn-6*, in *C. elegans* results in increased lifespan at 25 °C, but not at 20 °C. This overexpression also increases proteasome activity at 25 °C (Vilchez et al. 2012).

Exposure to high temperature or other stress in various organisms can elicit a highly conserved cellular mechanism, called the heat shock response (HSR), where rapid transcriptional activation is followed by expression of a group of chaperones, the heat shock proteins, which participate in protecting the cell against proteolytic stress (reviewed in Joutsen and Sistonen 2019). Upregulation of the HSR has not been detected upon exposure to 25 °C in *C. elegans* transcriptomics studies (Gómez-Orte et al. 2018; Schott et al. 2014), although an increase, albeit not at similar levels as in a typical HSR, in the fluorescence signal of a single-copy HSR reporter has been observed in transgenic animals grown from larval stage 1 (L1) until 1-day adults at 25 °C (Mendenhall et al. 2017).

Here we have examined the impact on proteasome function after exposing *C. elegans* to a mild stress of 25 °C for 1 day using both in vitro and in vivo approaches, and shown that changes in UPS-mediated protein degradation occur in a tissue-specific manner.

## Materials and methods

### *C. elegans* strains and manipulations

The following *C. elegans* strains were used: N2 Bristol, CL2070[*dvIs70[hsp-16.2p::GFP + rol-6(su1006)]*], SJ4005[*zcIs4[hsp-4p::GFP]*], SJ4100[*zcIs13[hsp-6p::GFP]*] (obtained from Caenorhabditis Genetics Center (CGC), Minneapolis, MN, USA); MAH215[sqIs11*[lgg-1p::mCherry::GFP::lgg-1 + rol-6]*] (Chang et al. 2017) (obtained from CGC); YD25[*xzEx25[vha-6p::Dendra2]*], YD27[*xzEx27[vha-6p::UbG76V::Dendra2]*] (Li et al. 2011); YD1[*xzEx1[unc-54p::Dendra2]*], YD3[*xzEx3[unc-54p::UbG76V::Dendra2]*] (Hamer et al. 2010); YD90[*xzIs1[vha-6p::UIM2::ZsProSensor]*] (Matilainen et al. 2013).

YD114[*xzIs2[unc-54p::UIM2::ZsProSensor]*] reporter strain was generated by injection of the plasmid unc-54*p*::UIM2::ZsGreen::MODC into N2 animals, followed by integration into the genome with gamma irradiation. The integrated line was outcrossed three times. The cloning of the plasmid has been described in Matilainen et al. (2013), as a precursor for strain YD90[*xzIs1[vha-6p::UIM2::ZsProSensor]*].

To generate YD115[*xzEx112[unc-54p::UIM2::GFP::MODC]*] reporter strain, GFP coding sequence was amplified with PCR from pS235 and cloned into the XhoI-NotI sites of unc-54*p*::UIM2::ZsGreen::MODC to replace the ZsGreen coding sequence. The plasmid was delivered into N2 animals by microinjection, and two independent lines were used in this study.

All strains were grown on standard NGM plates seeded with OP50 bacteria.

**Fig. 1:**
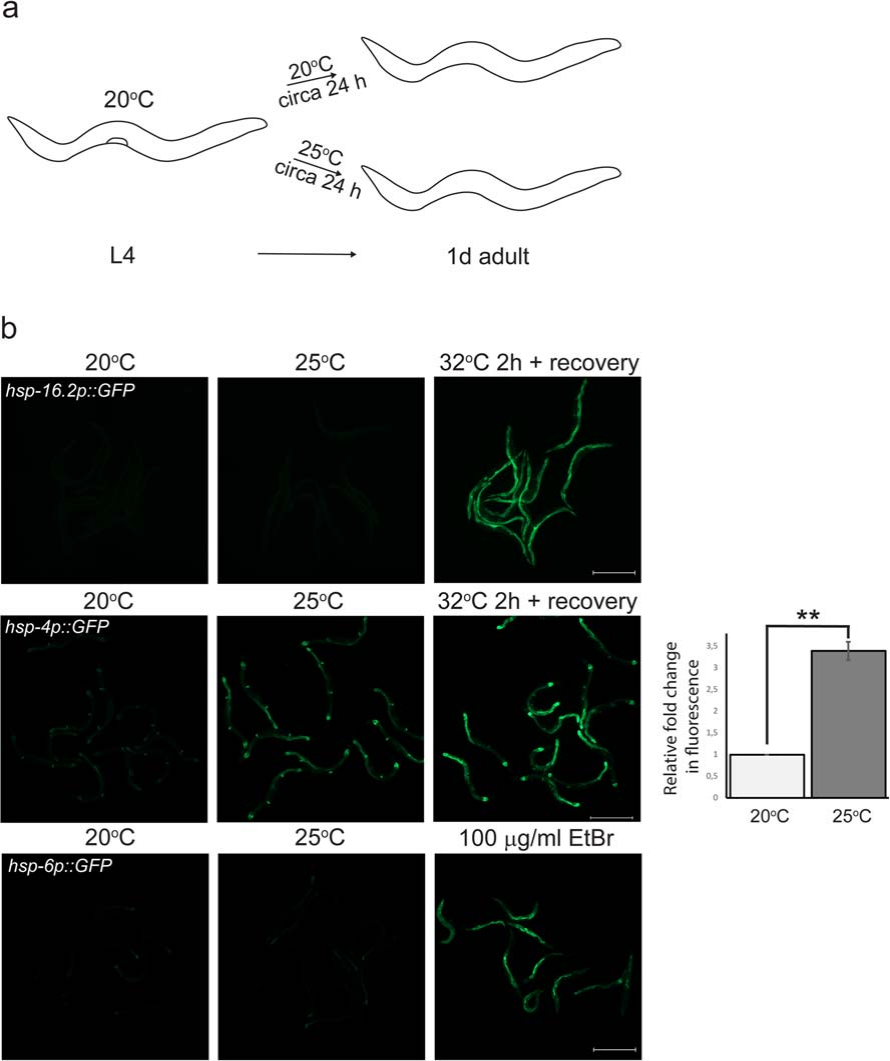
Differential stress responses at 25 °C. **a** Schematic drawing of experimental setup. **b** Exposure to 25 °C does not evoke an increased fluorescent signal with *hsp-16.2p::GFP* (HSR) (*n* = 25 animals per temperature) or *hsp-6p::GFP* (UPR^mt^) (*n* = 90) reporter strains. UPR^ER^ was activated upon exposure to 25 °C as shown with a threefold increased signal of *hsp-4p::GFP* reporter. Graph (on right) shows average fold change in UPR^ER^ reporter fluorescence intensity compared with 20 °C (set to 1), and is the mean of three independent experiments (*n* = 88). Error bar, SEM; **p value, < 0.01. Positive controls include treatment of 1-day adults for 2 h at 32 °C followed by a 4–7-h recovery period at 20 °C (*hsp-16.2p::GFP*, *hsp-4p::GFP*) and growing *hsp-6p::GFP* worms on OP50 containing 100 μg/ml EtBr. Scale bar, 500 μm

Unless otherwise stated, all experiments were started with larval stage 4 (L4) animals grown at 20 °C, either age-synchronised with bleach treatment or picked based on morphology. Half of these animals were transferred to 25 °C and half were left at 20 °C. All animals were assayed approximately 24 h later as 1-day adults (Fig. 1a).

For the experiments comparing intestinal polyubiquitin reporter animals grown continuously either at 20 °C or 25 °C, age-synchronised animals were imaged at 6–24-h intervals for 72 h starting from L3/L4. Data from three separate experiments was aligned by morphological criteria.

As positive controls for experiments with stress reporter strains, *hsp-16.2p::GFP* and *hsp-4p::GFP*, animals were placed as 1-day adults at 32 °C for 2 h and allowed to recover at 20 °C for 4–7 h. *hsp-6p::GFP* animals were grown for a few generations on NGM plates seeded with OP50 bacteria containing 100 μg/ml EtBr. Animals expressing bright fluorescence were picked for imaging.

### Quantitative real-time PCR

RNA was extracted from N2 animals using Nucleospin RNA kit (Macherey-Nagel) and converted into cDNA with Maxima First Strand cRNA synthesis kit (Thermo Scientific). Quantitative real-time PCR was performed with Maxima SYBR Green/ROX qPCR Master Mix (Thermo Scientific) and LightCycler 480 quantitative PCR machine (Roche) using an annealing temperature of 60 °C. *hsp-16.2* qPCR results were normalised to the geometric mean of three reference gene mRNA levels (*act-1*, *cdc-42*, *pmp-3*). Primer sequences were as follows: 5′-agatgtagatgttggtgca, 5′-tctcttcgacgattgcctgt (*hsp-16.2*), 5′-tcggtatgggacagaaggac, 5′-catcccagttggtgacgata (*act-1*), 5′-ctgctggacaggaagattacg, 5′-ctcggacattctcgaatgaag (*cdc-42*), 5′-gttcccgtgttcatcactcat, 5′-acaccgtcgagaagctgtaga (*pmp-3*).

### Microscopy and image analysis

Animals were anaesthetised with 1 mM levamisole, mounted on 3% agarose pads, and imaged as groups with a Zeiss Axio Imager wide-field light microscope and a Zeiss EC Plan Neofluar NA (numerical aperture) 10 × 0.3 objective, using the tiles function of the Zeiss Zen Blue software. For photoconversion of the tissue-specific UbG76VDendra2 and Dendra2 lines, green Dendra2 protein was converted to red using 405-nm UV light. Degradation of the red signal was followed 6 (intestine) or 24 (muscle) hours later. For analysis of autophagy reporter strain MAH215, animals were imaged with Zeiss LSM 880 confocal microscope and a Zeiss Plan Apochromat NA 63 × 1.4 objective.

Images were converted into tiff-format using Zen Blue and quantified with Fiji software. In brief, an original black and white tiles image was cut into a rectangle shape and background was subtracted using the corresponding command in the Fiji. Threshold was chosen so that mainly the fluorescent signal was selected and the same threshold was applied similarly to all images in the same experiment. The average mean fluorescent intensity was analysed by using the measure function in the Fiji. For quantification, no changes were made to the brightness of the images. For preparation of publication figures, the brightness of some of the images was increased in Adobe Photoshop, in an equal amount to corresponding images at 20 °C and 25 °C.

### In-gel proteasome assay and Western blotting

N2 animals were synchronised by bleach treatment and grown at 20 °C until L4 stage when half of the animals were placed at 25 °C. Both groups were collected the following day as 1-day adults with M9 buffer, pelleted, and frozen at − 80 °C.

For in-gel proteasome assay, pellets were lysed and the assay performed as previously reported (Matilainen et al. 2013) with the minor modification of running the gel first at 20 mA for 30 min and then at 40 mA for 2 h. Gel images were taken with MultiImage Light Cabinet using FluorChem 8900 software (Alpha Innotech Corporation). Quantifications were made with the Fiji software.

For Western blotting, animal pellets were placed in lysis buffer (50 mM Hepes, 150 mM NaCl, 5 mM EDTA), lysed by sonication, run on an SDS-PAGE gel, and immunoblotted with a Trans-Blot Turbo transfer system (Bio-Rad). To prevent deubiquitination and degradation of polyubiquitinated proteins, 20 mM N-ethylmaleimide (NEM) and 10 μm MG132 were added to the lysis buffer. The following antibodies were used: anti-20S proteasome alpha subunits 1-3, 5-7 (BML-PW8195, Enzo Life Sciences), FK1 for polyubiquitinated proteins (BML-PW8805, Enzo Life Sciences), and anti-alpha-tubulin (T5168, Sigma). ECL signals were visualised and quantified with Image Studio software (Licor). Alpha-tubulin signal was used for normalisation.

### Statistical analysis

For each experiment, the animals were imaged and quantified as groups of 4–15 animals, and an average of the mean fluorescent intensities of individual groups was calculated. The ratios of the average of control (20 °C) versus 25 °C animals in separate experiments were used to calculate the mean, standard error of the mean (SEM), and *p* value for each experimental setup. For calculating the *p* value, the Student *t* test (two-tailed) was used, except for Fig. S2b. When comparing fluorescent intensities of intestinal polyubiquitin reporter animals grown continuously either at 20 °C or 25 °C, the mean fluorescent intensities per group of animals were used as data points. For statistical comparisons, data points from time points 18 h, 24 h, 42 h, and 48 h were used. The *p* value was calculated with the Mann-Whitney test (exact, two-tailed).

A summary of animal/image/experiment numbers and *p* values relative to the 25 °C/20 °C ratio is presented in Supplementary Tables S1 and S2.

## Results

### Stress responses are differentially activated at 25 °C

To investigate the impact of a mild thermal stress on proteasome function, we exposed L4 stage *C. elegans*, grown at 20 °C, to 25 °C for 1 day. At first, we addressed whether this temperature shift may trigger an HSR by using the *hsp-16.2p::GFP* reporter strain, where GFP is expressed from an integrated chromosomal array under the promoter of a *C. elegans* gene orthologue of the small heat shock protein family. This reporter has previously been developed as a readout for HSR (Link et al. 1999). The reporter is highly induced in animals exposed to a 2-h heat shock at 32 °C (Fig. 1b), but the exposure to 25 °C for 1 day did not result in a detectable increase in fluorescence in the reporter animals (Fig. 1b). When we examined *hsp-16.2* mRNA levels by qPCR after the 1-day exposure at 25 °C, an increase was detected (mean fold induction 7.0 ± SD 5.3, *n* = 4). Although this indicates activation of the HSR, the level of induction is clearly less than the increase in *hsp-16.2* mRNA at 32 °C (mean fold induction 35 ± SD 16, *n* = 2). As the HSR might be transiently upregulated, we also examined the L4 *hsp-16.2p::GFP* reporter animals 2 h and 4 h after transfer to 25 °C, but did not detect any change in fluorescence (data not shown).

A temperature change can also activate other cellular stress pathways such as the unfolded protein response in the endoplasmic reticulum (UPR^ER^) or in the mitochondria (UPR^mt^). As indicators of ER or mitochondrial stress, we used, respectively, the well-established *hsp-4p::GFP* and *hsp-6p::GFP* reporter strains (Calfon et al. 2002; Yoneda et al. 2004). The intensity of the ER stress reporter was increased about threefold at 25 °C, suggesting activation of the UPR^ER^. On the contrary, the UPR^mt^ induction was not detected at 25 °C (Fig. 1b). We also tested for a transient UPR^mt^ upregulation by examining the *hsp-6p::GFP* reporter animals 2 and 4 h after transfer to 25 °C, and no increase in the fluorescence signal was detectable (data not shown).

### UPS activity is enhanced in the *C. elegans* intestine at 25 °C

For analysing the impact of a 1-day 25 °C exposure on the UPS, we utilised two different approaches: in vivo tissue-specific measurements of an ubiquitin-linked degradable fluorescence reporter and in vitro analysis of proteasome activity and levels in whole animal lysates.

**Fig. 2:**
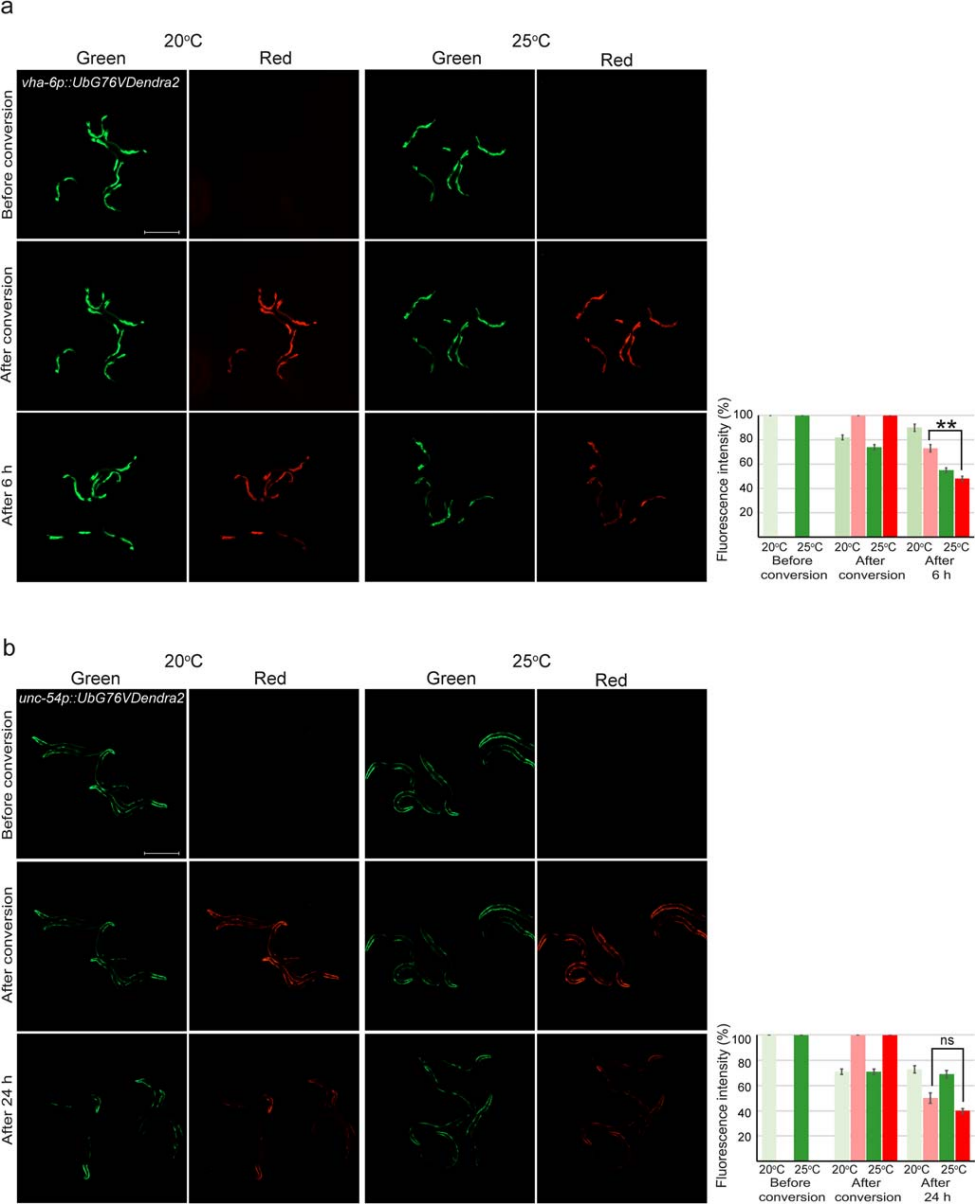
Enhanced UPS activity is observed in the intestine but not in the body wall muscle at 25 °C. Fluorescent micrographs and quantification of UbG76V-Dendra2 degradation in **a** intestinal and **b** body wall muscle cells at both 20 °C and 25 °C. Graph columns represent the average relative percentage of fluorescence prior to (green) or after (red) photoconversion, and are the average of a minimum of five independent experiments. Number of animals imaged is listed in Supplementary Table 1. Error bar, SEM; **p < 0.01; ns, not significant. Scale bar, 500 μm

For in vivo analysis of the UPS activity, we used our previously generated transgenic *C. elegans* expressing a proteasomal substrate consisting of a photoconvertible Dendra2 fluorescent protein linked to a non-cleavable ubiquitin moiety (UbG76V) in two different tissues, the intestine and body wall muscle cells (Hamer et al. 2010; Li et al. 2011; Matilainen et al. 2016). The Dendra2 protein can be irreversibly converted from a green to red fluorescent form with exposure to UV light. Green and red fluorescence are measured before and immediately after photoconversion, and after a follow-up time (Fig. 2). The decrease in red fluorescence intensity during the follow-up time is a relative measure of the degradation of the reporter and is not affected by the synthesis of new (green) reporter protein during the experiment. As we have previously shown (Hamer et al. 2010; Li et al. 2011; Matilainen et al. 2013), degradation of the UbG76V-Dendra2 reporter at 20 °C occurs faster in the intestine than in body wall muscle tissue, and therefore, 6- and 24-h end points, respectively, were selected for our experiments. When comparing the degradation of UbG76V-Dendra2 at 20 °C and 25 °C in the intestine, there is a clear increase in reporter degradation rate as the relative amount of remaining red fluorescence intensity in the intestine after 6 h was 48% at 25 °C compared with 73% at 20 °C (Fig. 2a). In body wall muscle, no major effect on reporter degradation was seen when comparing 20 °C and 25 °C (Fig. 2b). As such, the tissue-specific difference of slower degradation rate in body wall muscle compared with the intestine was maintained at 25 °C (Fig. 2). As controls, we used *C. elegans* expressing Dendra2 protein without UbG76V, which has been shown to be relatively stable throughout the follow-up time (Hamer et al. 2010; Li et al. 2011; Matilainen et al. 2013). In line with this, both at 20 °C and 25 °C, the control reporters were more stable than the UbG76V-Dendra2 reporters (Fig. S1). No difference in the Dendra2 fluorescence intensity was observed between 20 and 25 °C in the muscle cells (Fig. S1b), whereas a slight difference was detected in the intestinal cells (red fluorescence remaining after 6 h 82% at 25 °C compared with 94% at 20 °C) (Fig. S1a). Importantly, there is a significant difference between the degradation rate of UbG76V-Dendra2 and Dendra2 at 25 °C (*p* value < 0.01) (Table S1).

**Fig. 3:**
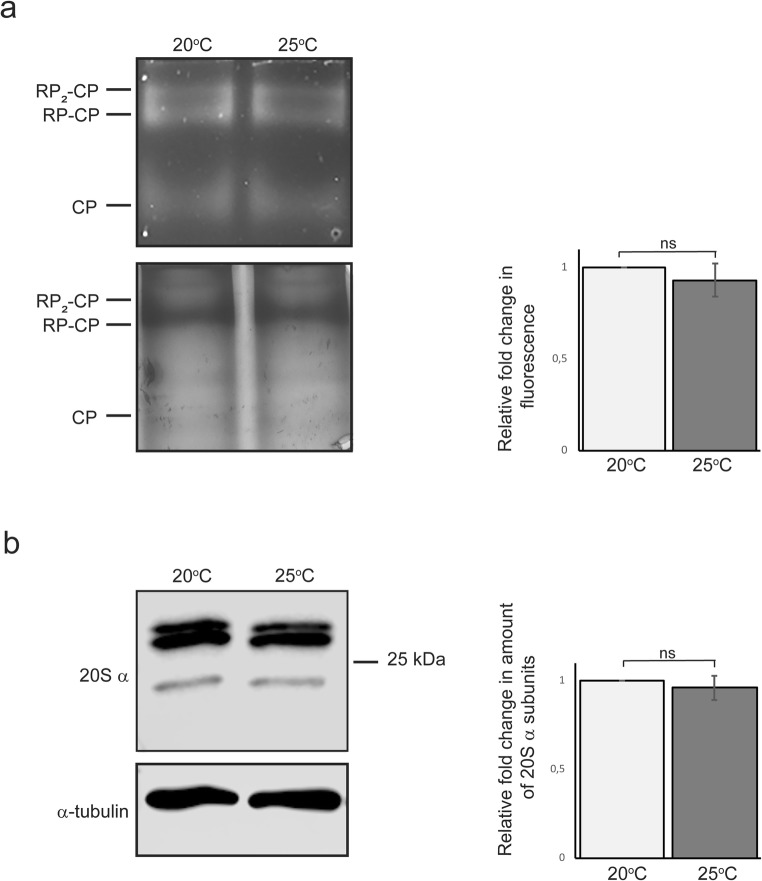
Proteasome catalytic activity and amount of 20S proteasome are unchanged in whole animal lysates of animals exposed to 25 °C. **a** In-gel proteasome activity assay (upper image), Coomassie staining of same gel (lower image) and quantification (right). **b** Western blot (left) and quantification (right) against proteasomal 20S alpha subunits. Anti-alpha-tubulin antibody was used as a normalisation control. Quantification graphs show the average fold change compared with 20 °C (set to 1) and are the average of 10 independent experiments. RP, regulatory particle; CP, core particle. Error bar, SEM; ns, not significant

As a complementary approach, the effect of a 1-day exposure of 25 °C on proteasome activity and amount was determined in vitro from whole *C. elegans* lysates. Lysates were run first on a native gel followed by an in-gel activity assay with fluorescent substrate for the chymotrypsin-like enzymatic activity of the proteasome 20S particle, and secondly on an SDS-PAGE gel followed by immunoblotting against 20S alpha subunits. In both cases, no difference between samples of animals from 20 or 25 °C was detected (Fig. 3), emphasising the importance of the use of tissue-specific assays.

### Polyubiquitinated proteins accumulate in body wall muscle at 25 °C

**Fig. 4:**
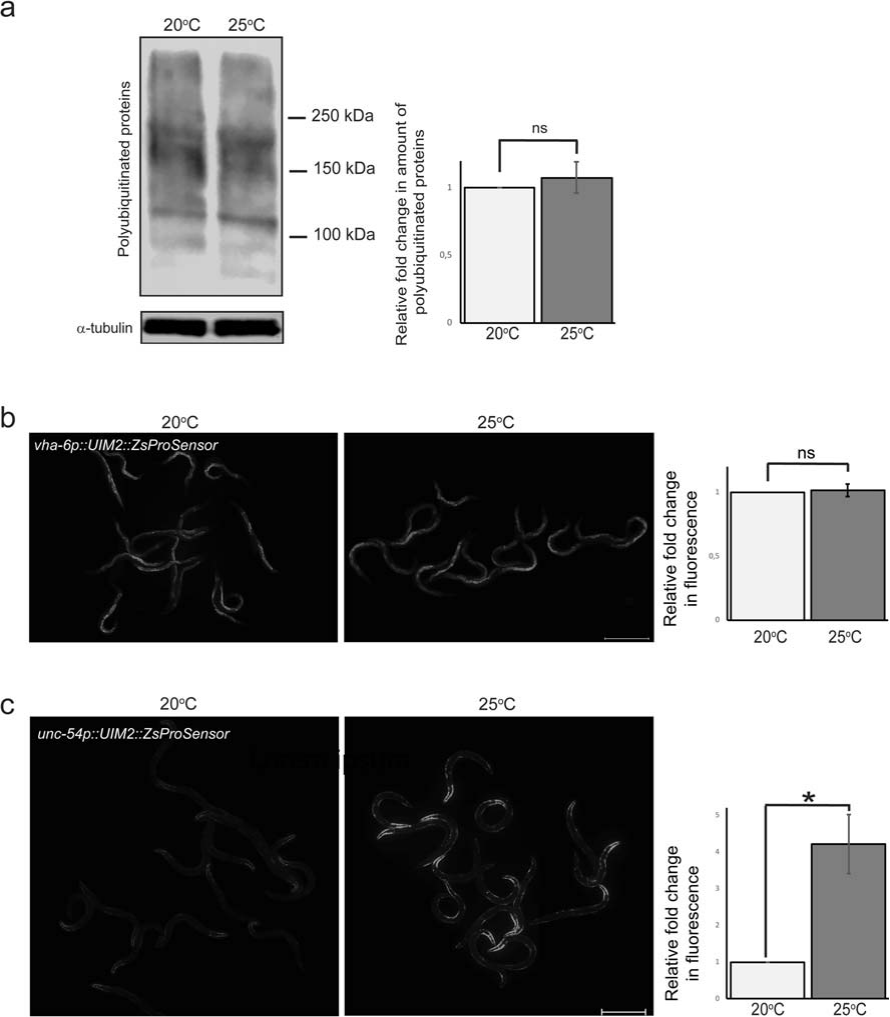
Polyubiquitinated proteins accumulate in body wall muscle at 25 °C. **a** Western blot against polyubiquitinated proteins (left) and quantification (right) of whole animal lysates of animals exposed to 20 °C or 25 °C. Anti-alpha-tubulin antibody was used as a normalisation control. Graph shows the average fold change compared with 20 °C (set to 1) and is the mean of 8 independent experiments. **b** No change is detectable in fluorescence intensity of the intestinal polyubiquitin reporter at 25 °C but **c** muscle reporter fluorescence increases approximately fourfold. Graphs (on right) show average fold change in fluorescence intensity compared with 20 °C (set to 1) and are the mean of a minimum of three independent experiments (*n* = 119 animals per temperature). UIM2 = ubiquitin-interacting motifs; ZsProSensor = ZsGreen::MODC transgene; MODC = C-terminal mouse ornithine decarboxylase. Error bar, SEM; ns, not significant; *p value, < 0.05. Scale bar, 500 μm

Accumulation of polyubiquitinated proteins can be a signal of dysfunctional protein degradation in the cell. We tested for this accumulation at 25 °C first by an in vitro approach using *C. elegans* lysates run on an SDS-PAGE gel and immunoblotted for polyubiquitin. No difference in accumulation of polyubiquitinated proteins between 20 and 25 °C was evident in the whole animal lysates (Fig. 4a).

To assess the accumulation of polyubiquitinated proteins in a tissue-specific manner in vivo, we utilised our previously published intestinal fluorescent transgenic reporter, consisting of ZsGreen fluorescent protein linked to ubiquitin-binding domains (UIM2) and a C-terminal mouse ornithine decarboxylase (MODC) degradation signal. This reporter has been shown to respond to proteasome inhibition and to co-localise with polyubiquitin-positive tissue immunostaining (Matilainen et al. 2013). Additionally for this study, we expressed this reporter in *C. elegans* body wall muscle cells. One-day exposure to 25 °C caused no increase in the intestinal reporter signal (Fig. 4b) but resulted in a clear upregulation of fluorescence in the body wall muscle cells (Fig. 4c). To assess this upregulation further, we generated a muscle polyubiquitin reporter in which the fluorescent protein ZsGreen of the ZsProSensor was changed to GFP (UIM2::GFP::MODC). Accumulation of fluorescent signal was also detected with this reporter, indicating that the increase in fluorescence was not fluorescent protein-dependent (Fig. S2a).

This data shows that a 1-day exposure to 25 °C increases accumulation of polyubiquitinated proteins in a tissue-specific manner, suggesting that maintaining protein homeostasis under a mild thermal stress may be more difficult in the body wall muscle compared with that in the intestine. However, accumulation of polyubiquitinated proteins does occur also in the intestine, if the animals are exposed to long-term growth at 25 °C (Fig. S2b). We grew the intestinal polyubiquitin reporter strain at 25 °C for several generations, and then imaged animals for 3 days starting from L4. This strategy was chosen to avoid any potential effects on fluorescence intensity resulting from growth rate differences between *C. elegans* cultured at 20 °C or 25 °C. Animals grown continuously at 25 °C displayed consistently higher levels of fluorescence compared with animals grown at 20 °C (Fig. S2b), suggesting a disturbance in proteasomal degradation capacity in the intestine at this growth temperature.

## Discussion

In this study, we show that a 1-day exposure to 25 °C has a tissue-specific impact on proteasome function in *C. elegans*. This temperature protocol was chosen first to assay proteasome function in 1-day old adults, comparable with previous studies with the tools in hand, and secondly as we wanted to avoid a developmental effect caused by the temperature increase. We show that the intestinal and body wall muscle tissue in *C. elegans* respond differently to a rise in ambient temperature. The relative degradation of a UPS substrate is increased by over 30% in the intestine (Fig. 2a) and concomitantly no accumulation of the polyubiquitin reporter is observed (Fig. 4b), suggesting that any potential changes in the amount of misfolded proteins caused by mild thermal stress can be overcome with increased proteasome activity. We also tested for any transient changes in the expression of the intestinal polyubiquitin reporter immediately after transfer to 25 °C by imaging the animals for 8 h at 2-h intervals (data not shown) and saw no systematic increase during this time. Thus, the intestinal protein degradation machinery is capable of immediately adapting to a temperature increase, instead of a delay causing temporary accumulation of polyubiquitinated proteins. In contrast, body wall muscle tissue does not significantly upregulate the UPS activity (Fig. 2b), possibly resulting in the detected accumulation of the polyubiquitin reporter fluorescence (Fig. 4c). These results, combined with in vitro analysis, further emphasise the importance of tissue-specific tools for studying physiological processes, as analysis of whole animal lysates may override subtle differences seen on tissue level. As such, our current results are in line with previous studies showing tissue-specific changes in the UPS activity and proteasome expression (Hamer et al. 2010; Li et al. 2011; Matilainen et al. 2013; Mikkonen et al. 2017).

Although research on the effects of ambient temperature changes on proteasome activity is scattered, the few studies available suggest that proteasome activity in vitro is reduced at rising temperatures in mice and killifish (Bartelt et al. 2018; Lu and Hsu 2015). It should be noted that also in the study by Bartelt et al. (2018), a tissue-specific response was reported as proteasomal activity decreased by increasing temperature in mouse brown adipose tissue but was either not affected or mildly increased in the liver, supporting the data we report here. In the study by Gómez-Orte and colleagues, expressions of *C. elegans* genes related to protein degradation were downregulated upon continuous growth at 25 °C (Gómez-Orte et al. 2018), in contrast to opposite results obtained in different species (Kristensen et al. 2016; Maor-Landaw et al. 2014; Traylor-Knowles et al. 2017). In this context, it is worth remembering that interpretation of proteasome transcriptional responses can be complicated by the so-called “bounce-back” effect where induction of proteasome subunit expression can be caused by inhibition of proteasome activity (Li et al. 2011; Meiners et al. 2003; Mikkonen et al. 2017).

In addition to the UPS, the autophagy-lysosome system also influences protein degradation, as both of these pathways utilise ubiquitinated substrates, and potential temperature-mediated disturbances of autophagy could affect the UPS. Early work in hepatocytes proposed that a rise in temperature increases autophagy in a linear fashion (Gordon et al. 1987), and later, it was showed in different tissues and cell lines that hyperthermia can induce autophagy (Bao et al. 2017; Ganesan et al. 2018; Zhao et al. 2009). Opposite results have been obtained in killifish muscle, where lowering the temperature increases autophagy linearly (Lu and Hsu 2015). A recent study by Chen and colleagues shows that exposing *C. elegans* L4 larvae to 15 °C for 1 day activates autophagy and molecularly mediates the extended lifespan observed at this temperature (Chen et al. 2019). However, when L4 larvae were shifted to 25 °C for 1-day, no difference in autophagy was witnessed by Chen et al. (2019) (transgenic GFP::LGG-1 reporter strain) or by us (data not shown, transgenic mCherry::GFP::LGG-1 reporter strain), suggesting that the autophagy-lysosomal pathway is not affected by a 1-day exposure to 25 °C.

We show that in *C. elegans*, the intestine appears to be more sensitive to a rise in temperature than the muscle tissue, suggesting better ability of the intestine to maintain proteostasis. In the muscle tissue, degradation of the UPS reporter is considerably slower compared with the intestine or neuronal cells (Hamer et al. 2010; Li et al. 2011). One possibility is that the protein quality control system in the intestine is more capable of quickly responding to stress. This could be caused by tissue-specific regulatory differences in, e.g., proteasome subunit translation or posttranscriptional regulation, regulatory interactors, substrate ubiquitination, or protein folding. As an example, we have previously shown that the deubiquitinating enzyme UBH-4 regulates proteasome activity differently in the intestine and muscle cells (Matilainen et al. 2013). Twenty-five degrees Celsius poses a mild stress upon *C. elegans* as indicated by a reduction in lifespan and progeny amount (Klass 1977), and by our results showing that HSR and ER stress are induced at least to some degree (Fig. 1b). However, it is not clear if there are tissue-specific differences in the stress responses upon a temperature increase. As the *hsp-4p::GFP* reporter is expressed and induced mainly in the intestine (Wormbase WS269 2019), it was not possible to compare the ER stress response in the *C. elegans* intestine and body wall muscle. Prolonged growth of intestinal polyubiquitin reporter animals at 25 °C showed increased fluorescent signal (Fig. S2b), indicating that the UPS system can be challenged also in the intestine, despite the initial upregulation of proteasome activity shown here. Unfortunately, we could not examine the intestinal UPS activity in animals grown for several generations at 25 °C as the UbG76VDendra2 transgenic line does not tolerate continuous growth at 25 °C.

In conclusion, our results reveal tissue-specific differences in *C. elegans* protein degradation by the UPS after a rise in an ambient temperature. Our results also emphasise a need for caution when interpreting data from experiments on various protein quality control systems and life span performed at 25 °C in *C. elegans*.

## Electronic supplementary material

Supplementary Fig. 1. Degradation of control Dendra2 in the intestine and body wall muscle at 25 °C. Fluorescent micrographs and quantification of Dendra2 degradation in **a** intestinal and **b** body wall muscle cells at both 20 °C and 25 °C. Graph columns represent the average relative percentage of fluorescence prior to (green) or after (red) photoconversion, and are the average of a minimum of 8 independent experiments. Number of animals is listed in Supplementary Table 1. Error bar, SEM; *p < 0.05; ns, not significant. Scale bar, 500 μm (PNG 621 kb).

High Resolution (TIF 63781 kb).

Supplementary Fig. 2 Muscle and intestinal tissue responses at 25 °C. **a***C. elegans* expressing polyubiquitin reporter with GFP in muscle show increased fluorescence at 25 °C. Quantification results (below) are from one independent line, similar results were obtained from a second line (data not shown). Graph shows average fold change in fluorescence intensity compared to 20 °C (set to 1), and is the mean of three independent experiments (n = a minimum of 40 animals per temperature). UIM2 = ubiquitin-interacting motifs; MODC = C-terminal mouse ornithine decarboxylase. Error bar, SEM; **p value, < 0.01. Scale bar, 500 μm. **b** Intestinal polyubiquitin reporter animals growing permanently at 25 °C show increased fluorescent signal compared to control animals at 20 °C. Animals were imaged at 6-24 h intervals for 72 h starting from L3/L4. The mean fluorescence intensity of the first time point at 20 °C was set at 1. Graph shows the average of three independent experiments. Error bar, SEM; p < 0.05 calculated using individual data points for time points 18 h, 24 h, 42 h, 48 h. L4, larval stage 4; YA, young adult; EL, egg-laying started (PNG 192 kb).

High Resolution (TIF 17136 kb).

Supplementary Table 1 Summary of number of experiments, images and animals used in one-day 25 °C experiments. (XLSX 11 kb).

Supplementary Table 2 Summary of number of experiments, images and animals used for statistical analysis of Fig. S2b. (XLSX 10 kb).
