# Supplementary figures and images for: Tissue-specific effects of temperature on proteasome function
Source: Cell Stress Chaperones. 2020 Apr 18;25(3):563–72. doi: 10.1007/s12192-020-01107-y (PMC7192876; doi:10.1007/s12192-020-01107-y)

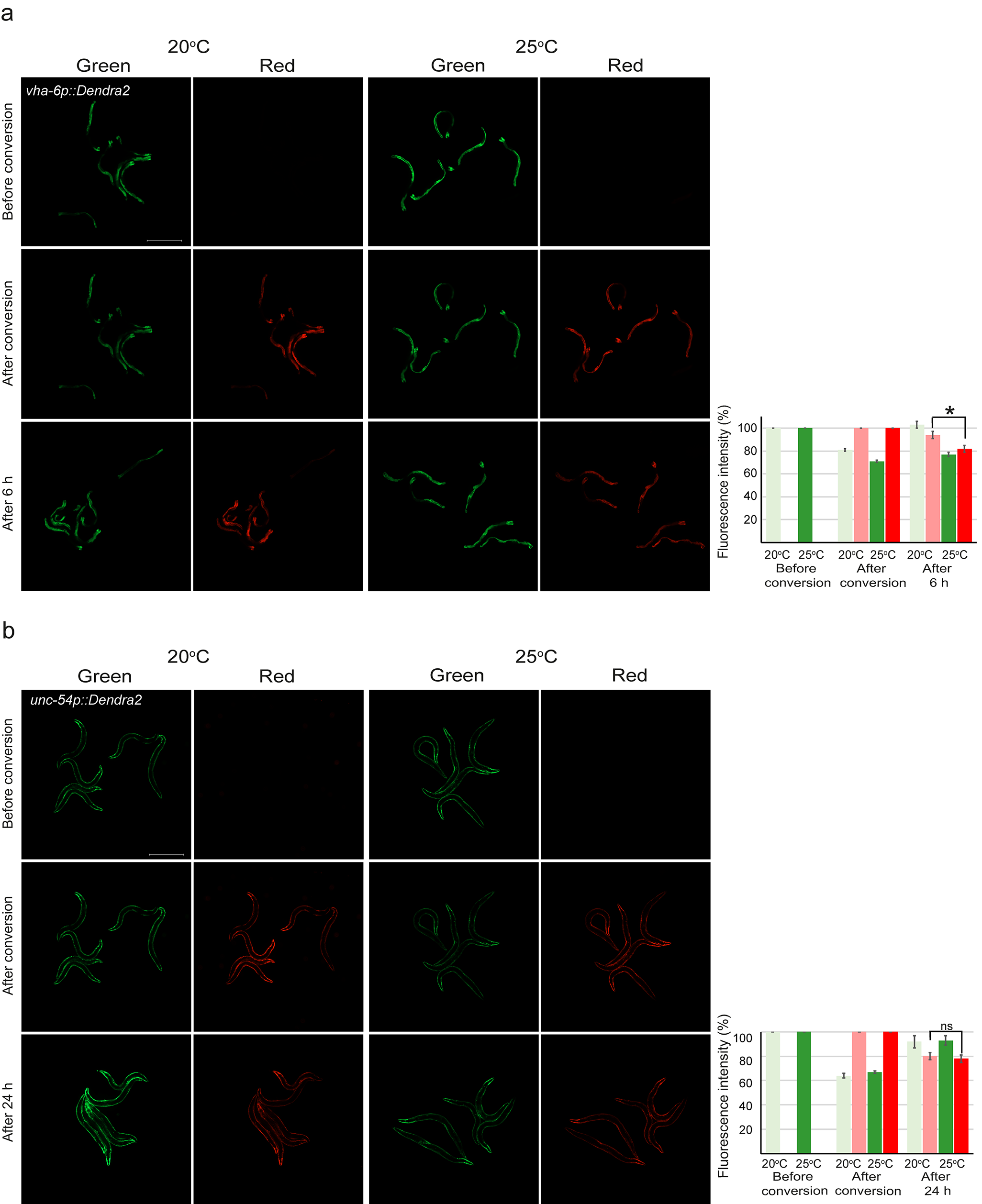

Supplement: Supplementary file 1 — Degradation of control Dendra2 in the intestine and body wall muscle at 25 °C. Fluorescent micrographs and quantification of Dendra2 degradation in a intestinal and b body wall muscle cells at both 20 °C and 25 °C. Graph columns represent the average relative percentage of fluorescence prior to (green) or after (red) photoconversion, and are the average of a minimum of 8 independent experiments. Number of animals is listed in Supplementary Table 1. Error bar, SEM; *p < 0.05; ns, not significant. Scale bar, 500 μm (PNG 621 kb). [file 12192_2020_1107_Fig5_ESM.png]

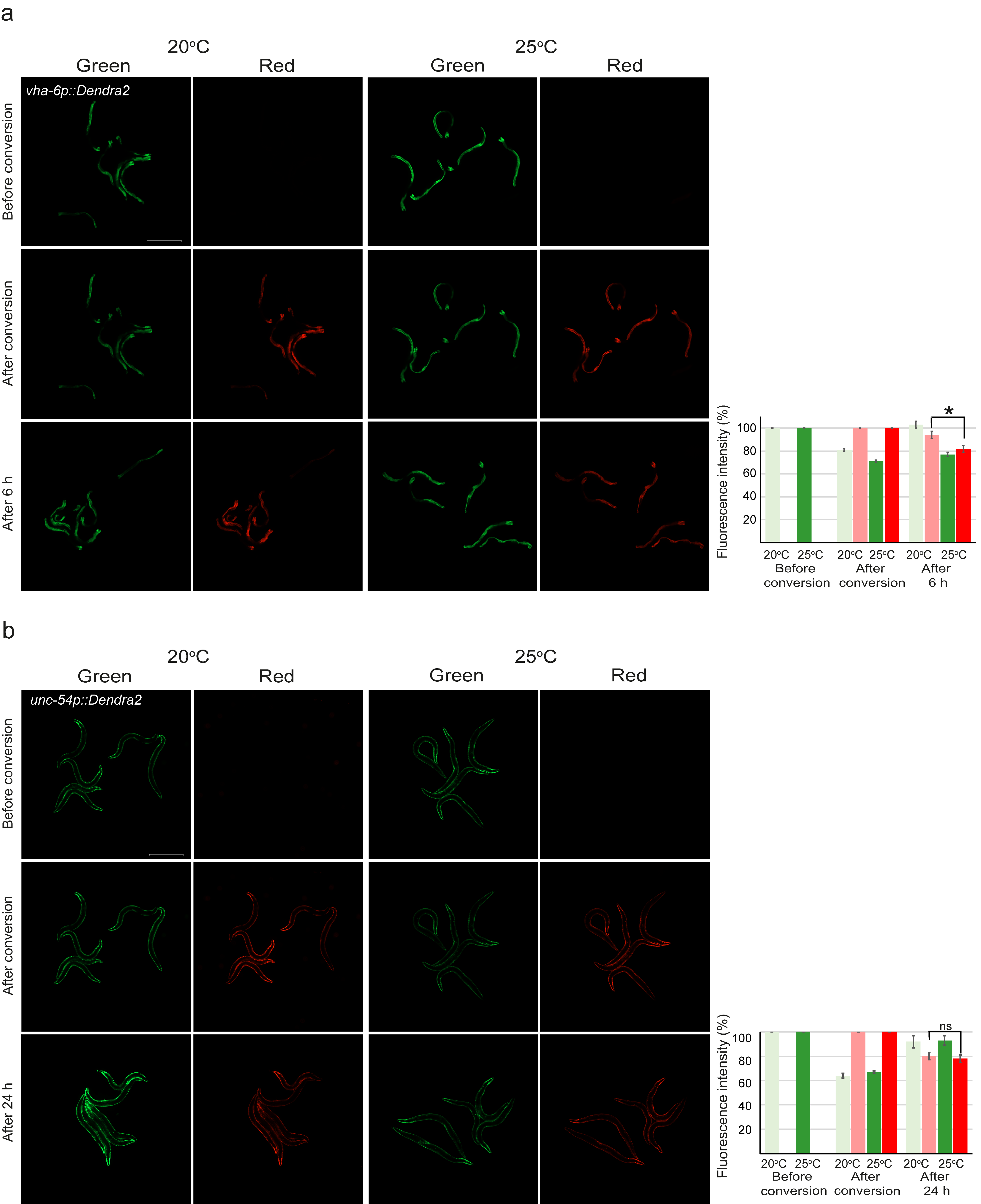

Supplement: Supplementary file 2 — High Resolution (TIF 63781 kb). [file 12192_2020_1107_MOESM1_ESM.tif]

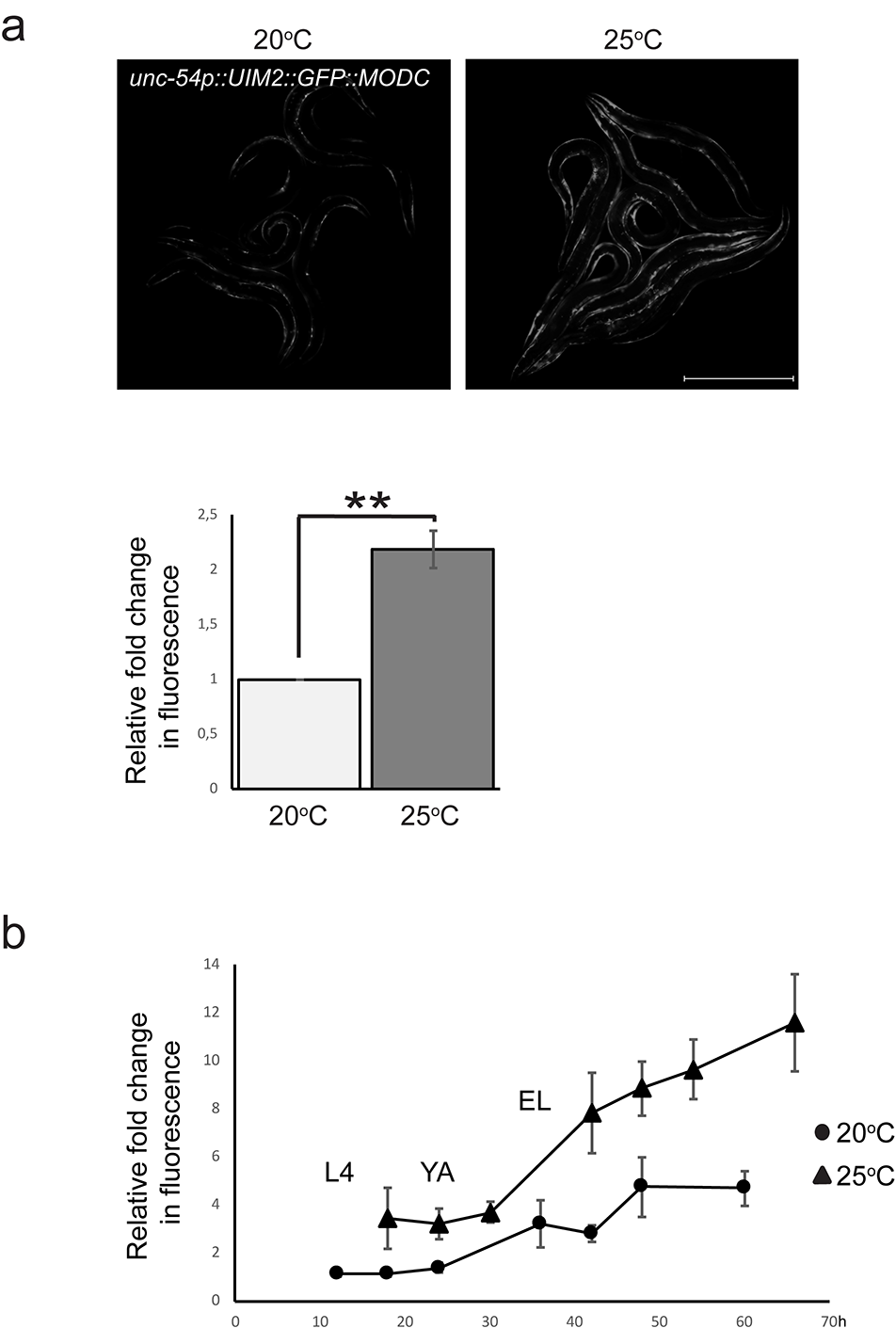

Supplement: Supplementary file 3 — Muscle and intestinal tissue responses at 25 °C. aC. elegans expressing polyubiquitin reporter with GFP in muscle show increased fluorescence at 25 °C. Quantification results (below) are from one independent line, similar results were obtained from a second line (data not shown). Graph shows average fold change in fluorescence intensity compared to 20 °C (set to 1), and is the mean of three independent experiments (n = a minimum of 40 animals per temperature). UIM2 = ubiquitin-interacting motifs; MODC = C-terminal mouse ornithine decarboxylase. Error bar, SEM; **p value, < 0.01. Scale bar, 500 μm. b Intestinal polyubiquitin reporter animals growing permanently at 25 °C show increased fluorescent signal compared to control animals at 20 °C. Animals were imaged at 6-24 h intervals for 72 h starting from L3/L4. The mean fluorescence intensity of the first time point at 20 °C was set at 1. Graph shows the average of three independent experiments. Error bar, SEM; p < 0.05 calculated using individual data points for time points 18 h, 24 h, 42 h, 48 h. L4, larval stage 4; YA, young adult; EL, egg-laying started (PNG 192 kb). [file 12192_2020_1107_Fig6_ESM.png]

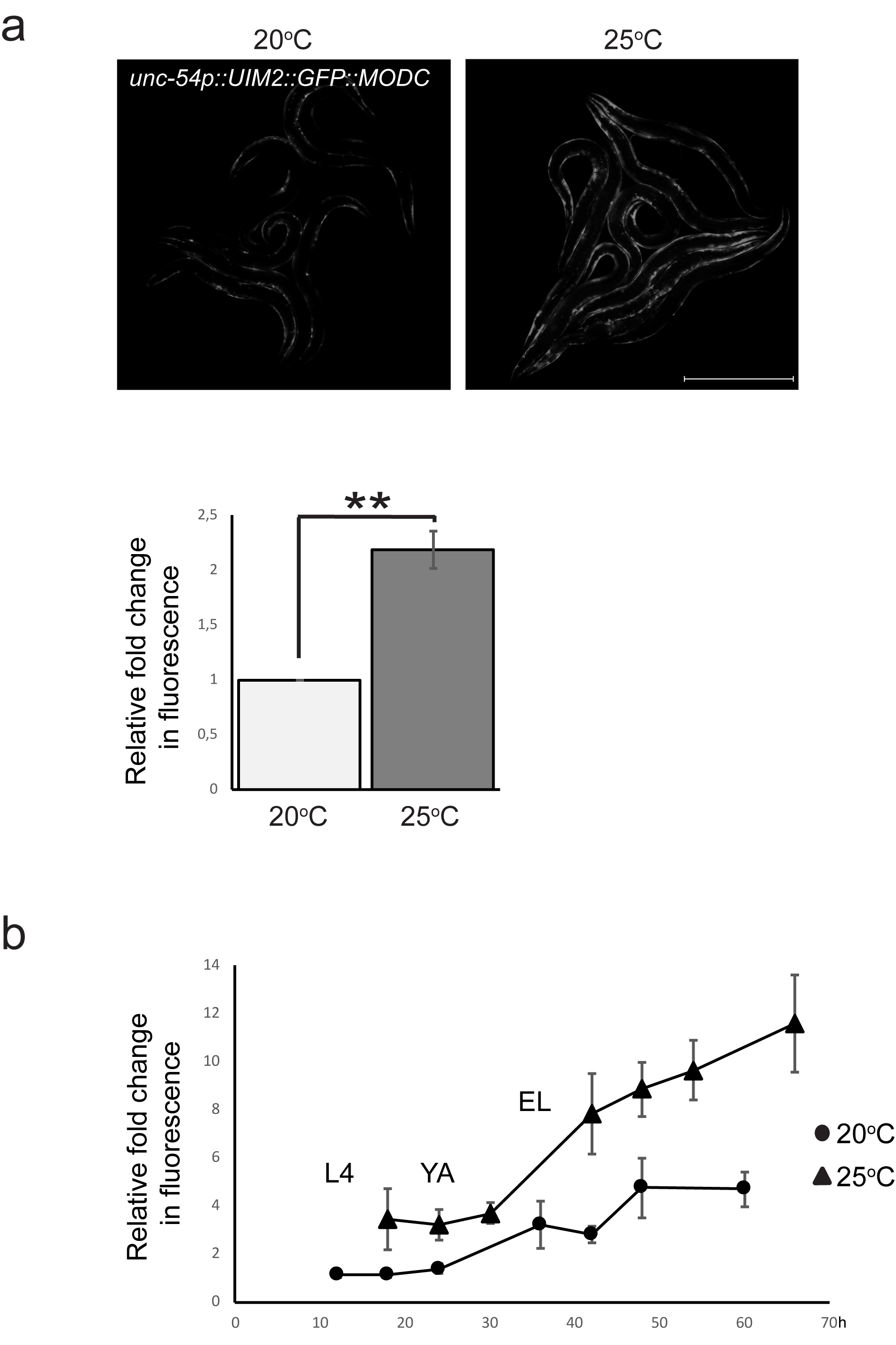

Supplement: Supplementary file 4 — High Resolution (TIF 17136 kb). [file 12192_2020_1107_MOESM2_ESM.tif]
